# Analysis of the functionally-narrowest portion of the pediatric upper airway in sedated children

**DOI:** 10.1097/MD.0000000000011365

**Published:** 2018-07-06

**Authors:** Ji-Hye Kwon, Young Hee Shin, Nam-Su Gil, Hyean Yeo, Ji Seon Jeong

**Affiliations:** Department of Anesthesiology and Pain Medicine, Samsung Medical Center, Sungkyunkwan University school of Medicine, Seoul, Korea.

**Keywords:** cricoid, computed tomography image, pediatric, subcricoid, trachea

## Abstract

The narrowest portions of the pediatric larynx are the glottis and subglottic region. However, the pliable and paralyzed subglottic region, acting like a curtain, is no resistance when passing an endotracheal tube. Therefore, the ‘functionally’ portion of the pediatric upper airway, which may be the most vulnerable to damage during intubation, is the unyielding portion below the cricoid cartilage. We investigated the functionally-narrowest portion below the cricoid cartilage.

Computed tomography (CT) was performed under deep sedation. CT images were used for measurement of dimensions and cross-sectional area (CSA) of the larynx at the level of the cricoid, subcricoid, and trachea. We analyzed the anteriorposterior (AP) diameter, transverse diameter, and CSA below the cricoid cartilage (at the cricoid, subcricoid, and tracheal levels).

CT images of 46 children from 8 months to 96 months were reviewed from electric medical record (EMR). The mean ± SD of AP diameter was the shortest at the subcricoid level (cricoid, 105.7 ± 15.8 mm; subcricoid, 94.6 ± 15.3 mm; and trachea, 101.5 ± 15.7 mm; *P* < .001). The mean ± SD of transverse diameter was the shortest at the trachea level (cricoid, 99.8 ± 12.2 mm; subcricoid, 102.5 ± 13.7 mm; and trachea, 98.8 ± 10.7 mm; *P* = .01). The mean ± SD of CSA was the smallest at the subcricoid level (cricoid, 8781.5 ± 1963.3 mm^2^; subcricoid, 8425.0 ± 2025.7 mm^2^; and trachea, 8523.7 ± 1791.1 mm^2^; *P* = .02). The AP diameter at the subcricoid level was narrower than the transverse diameter at trachea level (mean difference: 4.2 mm, 95% confidence interval [CI]: 0.7–7.7, *P* = .02).

Since the most susceptible portion for airway damage is unyielding portion, our findings suggest that, functionally, the narrowest portion of the pediatric larynx is located in the subcricoid region.

## 1 Introduction

In the pediatric population, identifying the narrowest portion of the upper airway has been one of the most critical issues for pediatric anesthesiologists because it could be the site with the highest potential for damage to the larynx in the intubated state.

The fact that the larynx is funnel-shaped, not cylindrical, has been commonly accepted knowledge ever since Bayeux described the pediatric airway based on anatomical sections of cadaveric larynxes.^[1,2]^ However, a recent study using advanced imaging modalities including magnetic resonance imaging (MRI) and computed tomography (CT) reported that the shape of the pediatric airway is a reverse cone shape, rather than a funnel shape and, the narrowest portions of the pediatric larynx are the glottis and subglottic region, rather than the cricoid cartilage.^[3–6]^ However, the pliable and paralyzed subglottic region, acting like a curtain, is no resistance when passing a endotracheal tube.^[7,8]^ Therefore, the ‘functionally’ portion of the pediatric upper airway, which may be the most vulnerable to damage during intubation, is the unyielding portion below the cricoid cartilage.

There is currently a lack of knowledge about the specific site of the functionally-narrowest portion of the airway below the cricoid cartilage in the pediatric population. We investigated the narrowest portion below the cricoid cartilage and the correlation between the narrowest diameter and demographic variables, including age, height, and weight.

## 2 Materials and methods

Our institutional review board approved this retrospective study (SMC 2017–06–066) and waived the requirement for written informed consent. All data were derived from our institution's electronic medical records (EMR) and picture archiving communication system (PACS). The records of patients with oncologic diseases (lymphoma, neuroblastoma, and leukemia) who underwent CT simulation before proton therapy between January 2014 and May 2017 were initially screened. Patients were free from airway symptomatology, and the presence of anatomical features which might interrupt assessment of airway imaging was determined by reviewing EMRs and the patients’ clinical courses. Exclusion criteria included patients with anatomical deformities of the larynx and any other condition or diagnosis that investigators felt would cause abnormal laryngeal anatomy, including the presence of a tracheoesophageal fistula, extra pulmonary or intrathoracic mass, diaphragmatic hernia, mediastinal mass, atelectasis, or genetic syndromes. Patients who received airway management with either an endotracheal tube or supraglottic airway, and those with a tracheostomy, were also excluded.

CT was performed under deep sedation. After taking an electrocardiogram, a non-invasive blood pressure, pulse oximetry monitoring, and pre-oxygenation with oxygen via facial mask were performed with the patient in the supine position. Propofol (effect site target concentration of 3.0–5.0 μg/mL) was continuously infused intravenously using a target-controlled infusion pump (Syramed μSP 6000, Acromed, Regensdorf, Switzerland) with a Paedfusor model during CT examination by the attending anesthesiologist. Spontaneous respiration was maintained throughout radiographic imaging without an airway device, endotracheal tube, or supraglottic airway. During CT examination, oxygen was administered at 4 L/min via a facial mask, and continuous nasal end-tidal carbon dioxide monitoring was performed. The patients were placed in the supine position with their necks extended.

The multidetector (MD)-CT scanner (GE Healthcare, Waukesha, WI) was used for radiological evaluation. Airway images were acquired from the vocal cords to the level of the carina. Thin sections of the patients’ airway were gained by helical CT imaging. The scanning technique was similar for all MD-CT scanners used. The image resolution was standardized for all CT scans, and a slice thickness of 2.5 mm was used for measurements. All images acquired included the airway extending from the vocal cords to the trachea.

**Figure 1:**
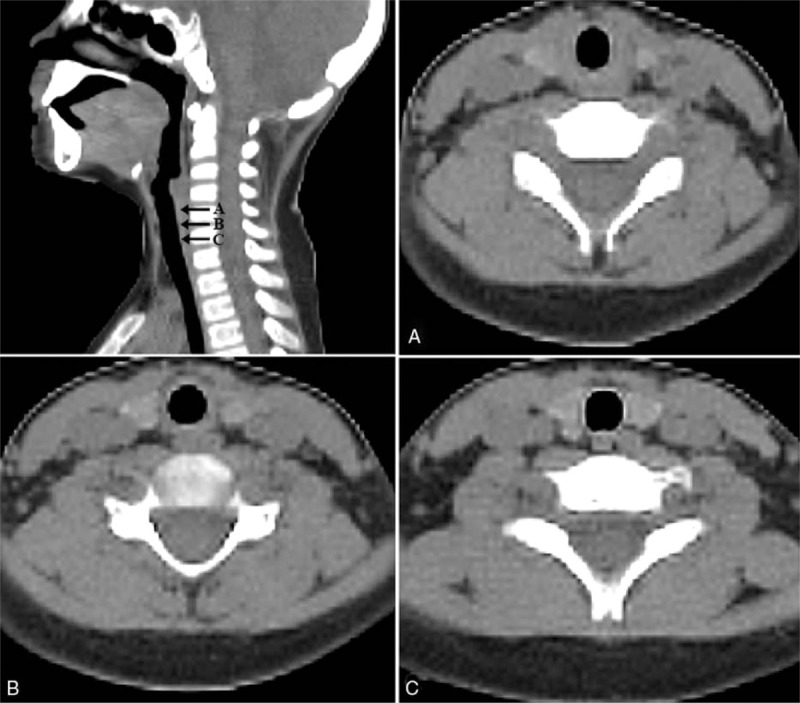
Sagittal image of the airway of a pediatric patient showing the transition from the subglottic level to the tracheal level. Axial image of the airway showing the cricoid level (A), subcricoid level (B), and trachea level (C).

CT images were used for measurement of dimensions and cross-sectional areas (CSAs) of the larynx at the level of the cricoid, subcricoid, and trachea. Axial scans were obtained from the oropharynx as the most cephalad end to the carina below the trachea as the most caudal end. Sagittal images were used as a reference to identify the proper levels (Fig. 1). The cricoid level was identified by using the complete cricoid cartilage as the radiological parameter. The subcricoid level was defined as the CT imaging slice immediately caudal to the cricoid ring. The standard soft tissue windows were used with window of 400 and level of 40 HY (400/40). In each patient, the anteroposterior (AP) and transverse diameters and CSA were measured respectively at 3 levels using axial scans. A single investigator uniformly produced all CT images. The investigator was blinded to patient demographics, including age, weight, and height, until measurements were finished. Electrical calipers were used for measurements.^[9,10]^

The primary purpose of this study was to evaluate the narrowest portion below the cricoid cartilage, which could be considered the functionally-narrowest portion in the pediatric larynx. We also aimed to investigate the correlation between the narrowest diameters and demographic variable including age, height, and weight.

## 3 Statistical analysis

Categorical variables were presented as the number (%) and continuous variables were presented as the mean ± standard deviation (SD). Interval data were analyzed at the below cricoid cartilage level (cricoid, subcricoid, and trachea levels), using the repeated-measure analysis of variance combined with Bonferroni correction for post-hoc testing. To obtain the narrowest diameter, the narrowest AP and transverse diameters were analyzed using paired *t* tests. Student *t* test was used to compare the demographic data, AP, transverse diameter, and CSA according to gender. The relationship of the functionally-narrowest portion below the cricoid level and age, height and weight was analyzed using linear regression analyses. All significant variables (*P* < .05) were entered into stepwise multiple linear regression procedures. A *P*-value of < .05 was considered to indicate a statistically significant result. Statistical analyses were performed using SPSS 22.0 (SPSS Inc., Chicago, IL).

## 4 Results

Data of 56 patients with CT images were retrieved from the EMR. Ten patients were excluded based on exclusion criteria, and the CT images of the remaining 46 patients were reviewed. Demographic characteristics are reported in Table 1.

**Table 1:**
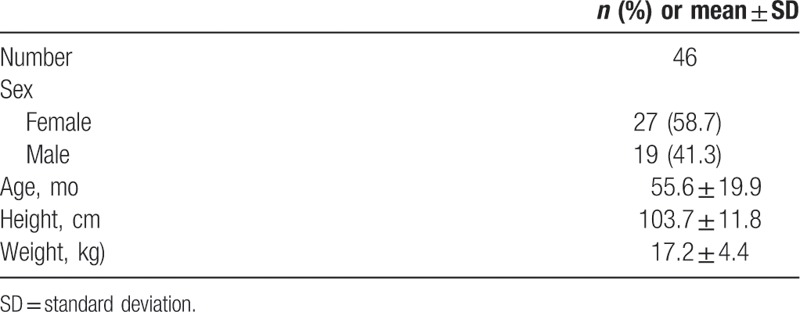
Demographic characteristics.

The AP diameter, transverse diameter, and CSA measurements at the cricoid, subcricoid, and trachea levels are reported in Table 2. The AP diameter was the shortest at the subcricoid level (mean ± SD: 94.6 ± 15.3 mm, *P* < .001). The transverse diameter was the shortest at the trachea level (mean ± SD: 98.8 ± 10.7 mm, *P* = .01). The CSA was the smallest at the subcricoid level (mean ± SD: 8425.0 ± 2025.7 mm^2^, *P* = .02). The AP diameter at the subcricoid level was narrower than the transverse diameter at trachea level (mean difference: 4.2 mm, 95% confidence interval [CI]: 0.7–7.7, *P* = .02).

**Table 2:**
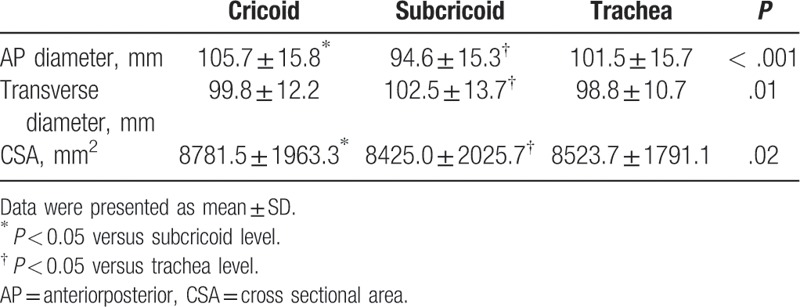
Measurements of the AP diameter, transverse diameter, and CSA at the cricoid, subcricoid, and trachea level.

**Figure 2:**
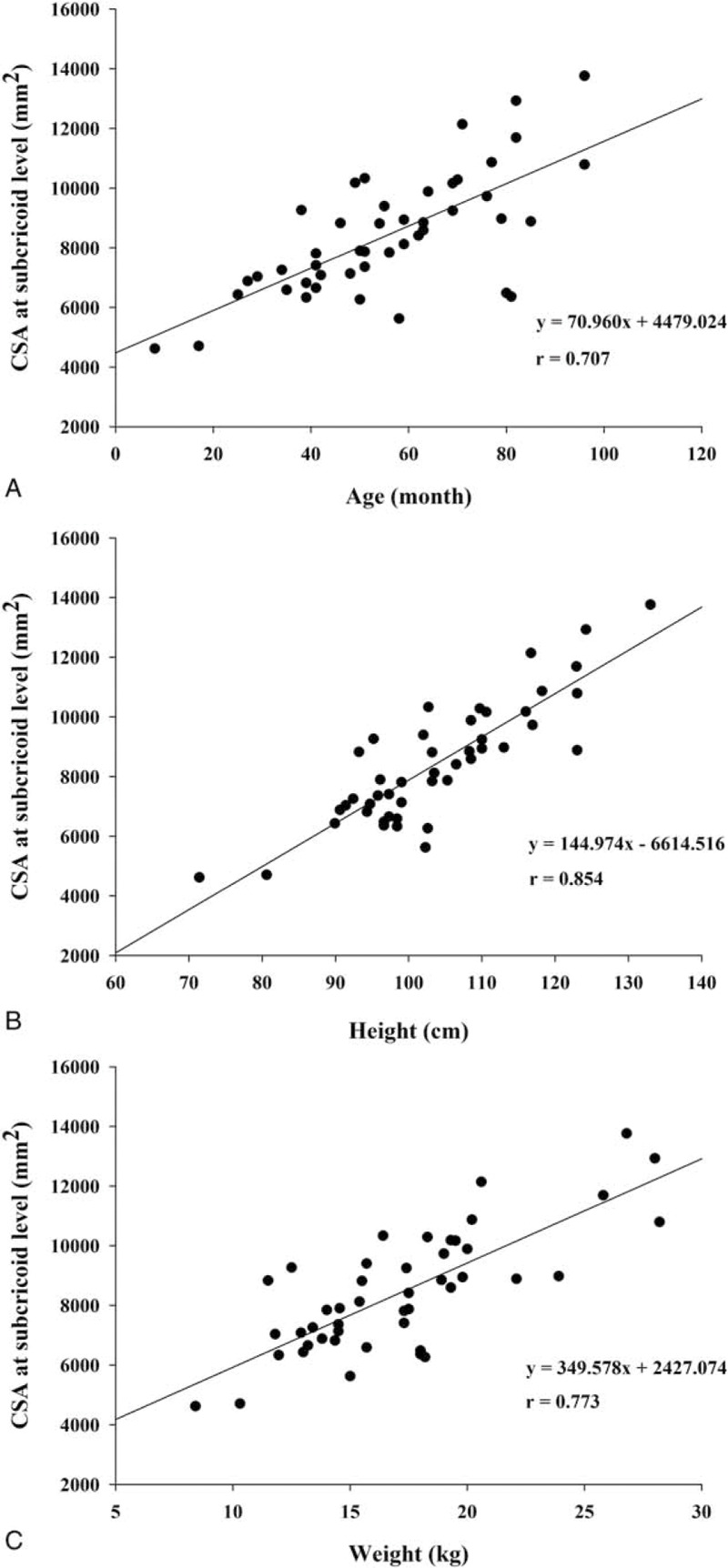
Correlation between the CSA at the subcricoid level and age (A), height (B), and weight (C). CSA = cross sectional area.

**Figure 3:**
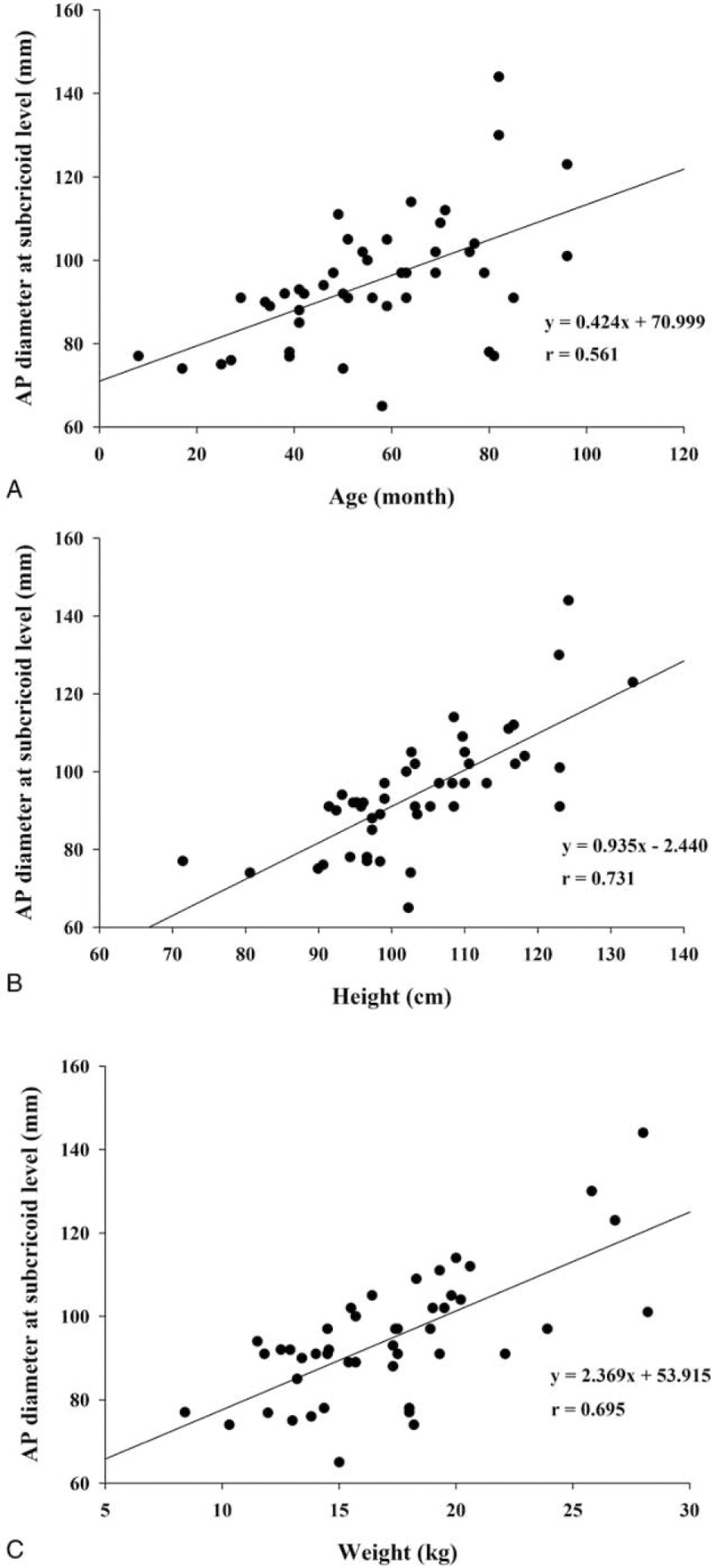
Correlation between the AP diameter at the subcricoid level and age (A), height (B), and weight (C).

Figure 2 shows the correlation coefficients and formulas comparing the relationships between the CSA at the subcricoid level and demographic data. The CSA at the subcricoid level showed the highest correlation with preoperative height, followed by weight and age (r = .854, *P* < .001; r = .773, *P* < .001; and r = .707, *P* < .001, respectively). And, Figure 3 shows the correlation coefficients and formulas comparing the relationships between the AP diameter at the subcricoid level and demographic data. The AP diameter at the subcricoid level showed the highest correlation with preoperative height, followed by weight and age (r = .731, *P* < .001; r = .695, *P* < .001; r = .561, *P* < .001, respectively). As shown in Table 3, we found that the age, height, and weight were significantly associated with AP diameter at the subcricoid level and CSA at the subcricoid level (*P* < .05). Therefore, these variable were entered into a stepwise multiple regression analysis, which revealed that height was significantly and positively correlated with AP diameter at the subcricoid level and CSA at the subcricoid level (B = 0.731, *P* < .001 and B = 0.854 *P* < .001, respectively). There was no statistically significant difference between boys and girls for the AP, transverse diameter and CSA of the cricoid, subcricoid, and trachea (Table 4). And, the apnea (> 10 sec) and desaturation (< 90%) were not observed during the CT examination with deep sedation.

**Table 3:**
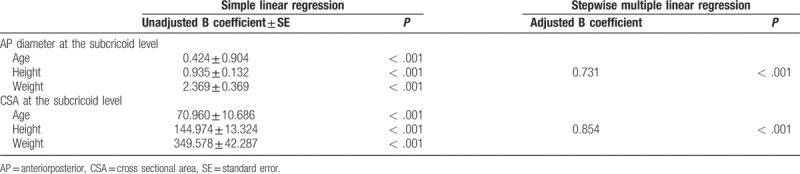
The relationship of the functionally narrowest portion and age, height and weight.

**Table 4:**
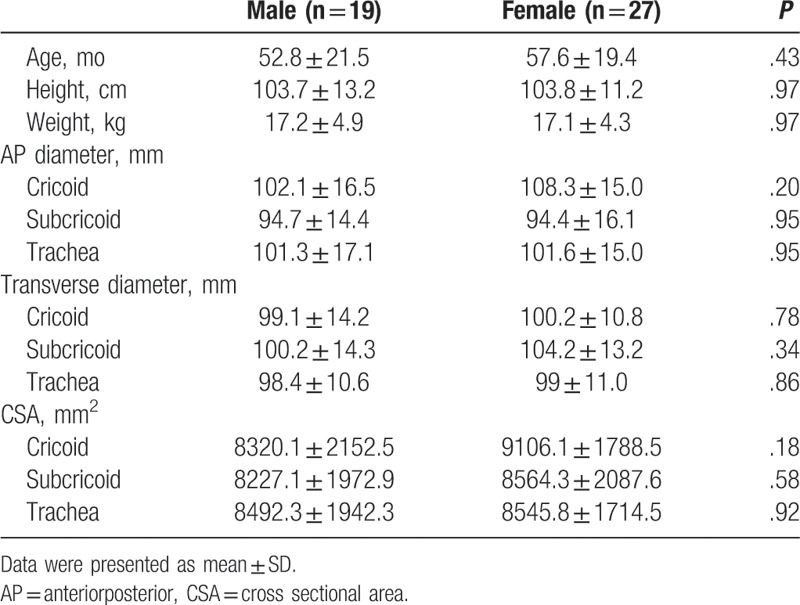
Demographic data and airway measurement according to gender.

## 5 Discussion

The major finding of our study was that the functionally-narrowest portion of the larynx in sedated children is the subcricoid level. And, we concluded that the functionally-narrowest diameter is AP diameter at the subcricoid level. Also, our study suggested that the correlation between the subcricoid level and patient height is greater than the association between this diameter and age or weight.

In the pediatric larynx, it has been revealed by several studies that the cross section of the airway is elliptical, not circular. As shown in Figure 1, the pediatric airway does not circular shape. However, the endotracheal tube (ETT) has a circular shape on cross-section.^[3,4]^ Because of this difference in shape, finding the smallest portion of the AP or transverse diameters is more important than finding the CSA, given that the elliptic larynx exerts more compression and ischemia on the smaller diameter than on the larger diameter. In our study, we focused on finding the smallest portion of the AP and transverse diameters. Recent studies have demonstrated that the narrowest portion of the pediatric airway is at the subglottic level, not the cricoid level.^[5,6]^ Our data are consistent with these studies. However, airway damage often occurs at the rigid, narrowest portion of the larynx. Several studies have addressed that the functionally and anatomically narrowest portion of the upper airway in children is around the cricoid cartilage, and that this site is important when considering the possibility of airway damage because of its rigid nature.^[8,11,12]^ It has also been confirmed in postmortem infants that the area around the cricoid cartilage is a commonly injured site in the pediatric larynx.^[11,12]^ The use of an ETT with a large external diameter can induce mucosal edema and injuries by pressure and friction.^[11,12]^ For that reason, if a pliable structure, such as the vocal cords or subglottic region, is regarded as the narrowest portion of the larynx, damage can be caused to the larynx if an inadequate ETT size is chosen for sealing the airway at this pliable region. In our study, the narrowest portion of the unyielding portion of the pediatric airway was found to be the AP diameter at the subcricoid level. And, the CSA was also the smallest at the subcricoid level. Therefore, the subcricoid level will be the most susceptible to laryngeal damage upon intubation. This should be considered in the intubation of pediatric patients.

We also analyzed the correlation between the functionally-narrowest portion of the airway and demographic variables, including age, height, and weight. A previous study reported that weight was the most predictive variable of airway diameters.^[13]^ In addition, other recent studies simply investigated the relationship between age and airway dimensions, without examining other variables.^[5,6]^ However, we have investigated the relationship between the narrowest airway dimension and age, height, and weight. Our findings suggest that height may be a more accurate predictive variable for the narrowest portion below cricoid cartilage area than age or weight. Therefore, a height based formula for the optimal ETT size may be more accurate than age and weight based formulas.

Finding the optimal ETT size for the pediatric population has been a critical issue for pediatric specialists, and many studies have suggested a wide variety of formulas.^[14,15]^ Recently, studies on using ultrasonography-based estimations of pediatric airways, rather than using one of these conventional formulas, for optimal pediatric ETT size selection have been published. These studies suggested that measuring the subglottic airway diameter with ultrasonography was a better option for predicting the optimal outer ETT diameter than standard age- and height-based formulas.^[16,17]^ However, estimations by sonography were done at the subglottic portion of the airway, and the formulas were also based on this area. In contrast, the AP diameter at the subcricoid level was the narrowest portion of the airway in our study. The sonography makes it easier to measure the diameter of the subcricoid level than MRI. Although further studies are needed to confirm the most likely site of damage when considering pathological and anatomical aspects, our results suggest that measurement by sonography at the subcricoid level may be helpful in choosing the optimal ETT size for children.

A slight head extension worsened the alignment of the pharyngeal and laryngeal axes, but improved alignment of the line of vision and the laryngeal axis.^[18]^ In our study, we was performed a slight head extension to improve ventilation in pediatric patients with sedation. This could have an influence on measurements and shape of larynx. However, we evaluated the rigid portion of the airway, which may have minimized this effect.

Our study had several limitations. First, this is a single center, retrospective study. And, our study populations included patients with oncological disease rather than healthy patients. Thus, there was a risk of selection bias. However, since the patients with airway problems were excluded from our study, the selection bias would not have had significantly affected the results of the current study. Second, although a reference sagittal image of the CT scans was used to accurately identify the levels of the airway, there could be a slight mismatch between the measured levels and actual levels depending on the patient's height or airway characteristics, because the slice thickness of the CT scans was constant at 2.5-mm intervals. Third, patients were sedated and unparalyzed with spontaneous respiration, and the phase of respiration was not controlled during the examination. Since airway dimensions can change depending on the respiratory phase (inspiration vs expiration), measurements done in different phases may be significantly difference. However, since we evaluated the rigid portion of the encircling cartilage, the impact of the respiratory phase on the airway dimensions is less than in other studies focusing on the vocal cord.

In conclusion, since the most susceptible portion for airway damage is unyielding portion, our findings suggest that, functionally, the narrowest portion of the pediatric larynx is located in the subcricoid region. Therefore, the subcricoid level can be predicted as a vulnerable site for airway damage during intubation. However, further studies about airway damage are needed to support our study. Also, there is high correlation between this functionally-narrowest portion and height. This finding will be help determine the optimal ETT size for intubation in pediatric patients.

## Author contributions

**Conceptualization:** Jihye Kwon, Ji S. Jeong.

**Data curation:** Hyean Yeo.

**Formal analysis:** Nam-Su Gil, Ji S. Jeong.

**Investigation:** Hyean Yeo.

**Methodology:** Young H. Shin, Nam-Su Gil, Hyean Yeo, Ji S. Jeong.

**Writing – original draft:** Jihye Kwon.

**Writing – review & editing:** Young H. Shin, Nam-Su Gil, Ji S. Jeong.
